# Formation of short chain fatty acids by the gut microbiota and their impact on human metabolism

**DOI:** 10.1080/19490976.2015.1134082

**Published:** 2016-03-10

**Authors:** Douglas J. Morrison, Tom Preston

**Affiliations:** Scottish Universities Environmental Research Centre, University of Glasgow, East Kilbride, Scotland

**Keywords:** gut microbiota, glucose homeostasis, host metabolic health, inflammation, short chain fatty acids

## Abstract

The formation of SCFA is the result of a complex interplay between diet and the gut microbiota within the gut lumen environment. The discovery of receptors, across a range of cell and tissue types for which short chain fatty acids SCFA appear to be the natural ligands, has led to increased interest in SCFA as signaling molecules between the gut microbiota and the host. SCFA represent the major carbon flux from the diet through the gut microbiota to the host and evidence is emerging for a regulatory role of SCFA in local, intermediary and peripheral metabolism. However, a lack of well-designed and controlled human studies has hampered our understanding of the significance of SCFA in human metabolic health. This review aims to pull together recent findings on the role of SCFA in human metabolism to highlight the multi-faceted role of SCFA on different metabolic systems.

## Introduction

Short chain fatty acids (SCFA) are the primary end-products of fermentation of non-digestible carbohydrates (NDC) that become available to the gut microbiota. They represent the major flow of carbon from the diet, through the microbiome to the host. The discovery that SCFA appear to be the natural ligands for free fatty acid receptor 2 and 3 (FFAR 2/3), found on a wide range of cell types, including enteroendocrine and immune cells, has led to renewed interest in the role of SCFA in human health.^1-3^ The link between dietary intake, gut microbiota diversity and function and their significance to human health is an active area of research at the present time. This reflects: 1) long-standing epidemiological evidence linking long-term high-fiber diets to improved health outcomes, 2) more recent observations from metagenomics studies on variations in gut microbiome diversity in metabolic disease and 3) improvements in our understanding of the intricate molecular signaling (referred to as “cross-talk”) between the gut microbiome and the host. Together, these represent a new frontier in our understanding of the determinants of major diseases in Western Societies. This is in part driven by the potential to intervene over the life-course with relatively simple and cost-effective interventions. However, at the present time, there is insufficient evidence to inform appropriate, evidence-based clinical or public health interventions with clearly defined outcomes using SCFA formulations. This review aims to examine the recent evidence around the role of SCFA as key signaling molecules between the gut microbiome and host health and bring together an integrated view of the role SCFA in human metabolic health.

## SCFA production by the gut microbiota

SCFA are produced mainly through saccharolytic fermentation of carbohydrates that escape digestion and absorption in the small intestine and the pathways of SCFA production are relatively well understood^4^ and recently described in detail.^5^ The major products are formate, acetate, propionate and butyrate. Lactate is also a major organic acid produced from the fermentation of selected, often rapidly fermentable NDCs.^6^ Relatively minor amounts of branched chain fatty acids are also produced, mainly through fermentation of protein-derived branched chain amino acids.^7^ Amino acid fermentation may also contribute to SCFA, mainly via acetate and propionate production. Relatively little is known about the role of formate in the gut. It has been linked to methanogenesis and appears to be elevated in inflammatory conditions.^8,9^ Lactate can also be further metabolised to acetate, propionate and butyrate by a number of cross-feeding organisms.^10-12^

Metagenomic approaches have facilitated characterization of bacteria responsible for SCFA production. Acetate production pathways are widely distributed among bacterial groups whereas pathways for propionate, butyrate and lactate production appear more highly conserved and substrate specific. For example, propionate production although distributed across a number of phyla is dominated by relatively few bacterial genera.^13^ Species such as *Akkermansia municiphilla* have been identified as key propionate producing mucin degrading organisms.^14^ On the other hand, deoxy-sugars such as fucose and rhamnose are particularly propiogenic because of metabolic pathways present to reduce the carbon skeleton, via the intermediate 1,2-propanediol, in select organisms.^13^ Fermentation of resistant starch is thought to contribute significantly to butyrate production in the colon and is dominated by *Ruminococcus bromii*, such that absence of the organism significantly reduces resistant starch fermentation.^15^ A surprisingly small number of organisms, dominated by *Faecalibacterium prausnitzii, Eubacterium rectale, Eubacterium hallii and R. bromii*, appear to be responsible for the major fraction of butyrate production.^16^ The link between diet, microbiome composition and SCFA production, although relatively well characterized is rather more difficult to predict and has often relied on *in vitro* fermentation data and animal models. *In silico* modeling of the complex dynamic relationships between dietary substrate, microbiota composition and substrate production holds promise enabling predictions of SCFA production from diet-gut microbiome interactions.^17^ However, high-level evidence from controlled human trials supporting SCFA as key regulation factors in human metabolism is largely lacking and there is significant reliance on associative studies, rather than interventional studies.

The field has been hampered by a lack of methodology to measure SCFA production directly in human studies, although recent work suggests that stable isotope techniques may hold promise.^18^ Observations in humans have largely relied on the measurement of stool SCFA output, although it is unclear whether stool SCFA output is a suitable proxy for luminal SCFA production.^19^ However, there is emerging evidence that diet-driven changes in microbiota diversity lead to variations in SCFA. In a recent diet-switch study, where African Americans were fed a high-fiber, low-fat African-style diet and rural Africans a high-fat, low-fiber western-style diet, the investigators observed profound shifts in gut microbiota composition, and SCFA and bile acids in the faecal water.^20^ A shift toward the butyrate producing organisms *Roseburia intestinalis, Eubacterium rectale* and *Clostridium symbiosum* along with increased butyrogenesis was observed on low-fat, high fiber feeding. Increases in CD3^+^ intra-epithelial lymphocytes and CD68^+^ lamina propria macrophages were also observed on high fat, low fiber diets suggesting increased inflammation in the absence of saccharolytic breakdown of fiber. Whether these changes translate into long-term impacts on host metabolism require intervention studies of longer duration. Changes in the microbiota of patients with inflammatory bowel disease (IBD) have been linked with decreased bacterial diversity and a loss of butyrate producing organisms such as *F. prausnitzii*.^21,22^ A similar loss of diversity has been observed in other auto-immune pathologies, such as psioaritic arthritis, suggesting a role for the microbiota and its metabolites in immune regulation.^23^ Insight into the links between diversity and function are also observed in interventions that have profound impacts on the diet - gut microbiome axis. Roux-en Y (RYGB) gastric bypass surgery led to enrichment of Bacteroidetes, Verrucomicrobia, and Proteobacteria at the phylum level and relatively greater propionate and lower acetate production suggesting that the gut microbiota contribute to reduced host weight and adiposity after RYGB surgery.^24^ These studies highlight the interdependence between diet (substrate), the gut microbiota and host metabolism and that, changes in SCFA and the microbiota are at least associated with profound effects on host metabolism.

## Site of SCFA production and biological gradient from gut lumen to the periphery

It is important to consider the site of SCFA production and the biological gradient across the various down-stream tissues to fully understand the biological effects of SCFA in humans. This is particularly pertinent for the translation of findings from animal studies which often utilize oral SCFA supplementation or high dietary fiber supplementation to induce changes in SCFA production. Oral SCFA are rapidly absorbed and oxidised, best exemplified by the use of sodium ^13^C-acetate as a tracer for liquid phase of gastric emptying.^25,26^ High circulating concentrations of SCFA (>1mmol/L; normal range, 0–5 μmol/L for propionate and butyrate), other than acetate, are observed in acidaemic disease in humans and have profound impacts on metabolism because of the toxicity of these organic acids at high concentrations.^27^ Whether oral SCFA feeding studies that lead to high circulating SCFA concentration in animals, particularly propionate, represent an appropriate model for human SCFA physiology requires further validation. Animal studies which use dietary fiber supplementation to manipulate colonic SCFA tend to use relatively high fiber supplementation; 5 – 20% w/w NDC in animal dry matter intake (DMI). Using the UK National Diet and Nutrition Survey (NDNS), estimates of human daily DMI (sum of protein, total fat, total carbohydrate, micronutrients and vitamins) can be obtained.^28^ Mean daily DMI from the NDNS survey in the UK for men and women (aged 19–64) is calculated at 418.3 g/d and 326.3 g/d respectively. Thus translating the fiber supplementation rates from animal experiments, a daily fiber supplementation in the range 20.9 – 83.7 g/d for men and 16.3 – 65.3 g/d of dietary fiber would be broadly comparable with the 5 – 20% w/w diet loadings used in animal studies. Also from UK NDNS data, mean dietary fiber intake in the UK diet has been measured at 14.7 g/d for men (aged 19–64) and 12.8 g/d for women (aged 19–64) respectively (measured as non-starch polysaccharide). Even the lowest supplementation levels used in animal studies represent a comparable substantial increase above habitual dietary fiber intake in humans.

**Figure 1.**
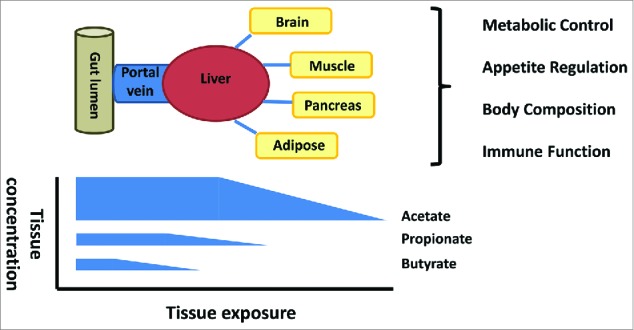
The gut lumen is the major site of production but the concentration gradient falls from the lumen to the periphery with selective uptake of butyrate at the epithelium, propionate at the liver and acetate in the periphery. The significance for host physiology of this biological gradient is poorly understood.

There is a strong biological gradient for each SCFA from the gut lumen to the periphery which leads to differing cell and tissue SCFA exposure (Fig. 1). The seminal work in sudden-death victims was first to highlight the significant reduction in butyrate, relative to acetate and propionate across the gut epithelium and also the significant reduction in propionate relative to acetate across the liver in humans.^29^ This has also been observed more recently with stable isotope flux studies in man where hepatic capacity to utilize SCFA balances gut SCFA production, leading to non-significant splanchnic propionate and butyrate output.^30,31^ These observations suggest that the roles of SCFA should be considered in each cell or tissue type within this biological gradient. The interplay between epithelial utilization and integrity, splanchnic utilization and peripheral availability requires delineation to determine whether increased production of all SCFA, or selective increases in individual SCFA at specific tissues, determines some of the observed metabolic effects.

## SCFA and gut integrity

It is well established that SCFA, and butyrate in particular, are important substrates for maintaining the colonic epithelium. Butyrate is the preferred fuel utilised by coloncytes and the primary site of butyrate sequestration in the body is the gut epithelium.^31,32^ Butyrate appears to have a dual role, sometimes referred to as the “butyrate paradox” whereby it induces proliferation in healthy colonocytes but terminal differentiation and apoptosis in transformed cells.^33,34^ However, in a mouse model of colorectal cancer, butyrate appears to fuel hyper-proliferation in MSH2 deficient colon epithelial cells leading to enhanced tumor formation.^35^ SCFA also appear to play an important role in regulating the integrity of the epithelial barrier through co-ordinated regulation of tight junction proteins (TJP) which themselves regulate the intracellular molecular highway between the lumen and hepatic portal system. Increased permeability is associated with translocation of bacteria and/or their cell wall components which trigger an inflammatory cascade that has been associated with obesity and insulin resistance.^36^ Increased bacterial lipopolysaccharide (LPS) triggers a toll-like receptor 4 (TLR4) mediated pro-inflammatory cascade in immune cells (monocytes, macrophages and Kupffer cells), leading to the activation of downstream signaling pathways, such as nuclear factor kappa β (NF-kΒ) and mitogen-activated protein kinase (MAPK), which can lead to inflammation driven by cytokines such as TNF-a and IL-6.^37^ Of the SCFA produced in the colon, butyrate appears to be the most important regulator of TJP and has been shown to enhance intestinal barrier function through increased expression of claudin-1 and Zonula Occludens-1 (ZO-1) and occludin redistribution; proteins which are critical components of the tight junction assembly.^38^ Butyrate has been shown to reverse the aberrant expression of ZO-1 and decrease LPS translocation leading to inhibition of macrophage activation, pro-inflammatory cytokine production and neutrophil infiltration resulting in reduced hepatic liver injury in rats.^39^ Further work is urgently needed in human models to determine whether SCFA play an important role in mucosal maintenance and integrity.

## SCFA and glucose homeostasis

A potential regulatory role for SCFA in glucose homeostasis, mediated through FFAR 2/3, has led to significant interest in pharmacological interventions targeting this receptor mediated pathway in metabolic disease.^40^ In the liver, propionate is gluconeogenic while acetate and butyrate are lipogenic.^41^ From stoichiometric equations of daily SCFA production from dietary fiber intake,^42^ daily propionate production, estimated to be 29.5 mg/kg/day for an average 85 kg human, is likely to make only a relatively small contribution to endogenous glucose production (2.2 mg/kg/min^43^) of which approximately 50% may be attributable to gluconeogenesis.^43^ The potential role of SCFA as signaling molecules regulating hepatic glucose homeostasis however has not been fully elucidated in humans. Acetate, propionate and butyrate appear to regulate hepatic lipid and glucose homeostasis in an adenosine monophosphate-activated protein kinase dependent manner involving peroxisome proliferator-activated receptor-γ regulated effects on gluconeogenesis and lipogenesis.^44^ Recently, evidence has also emerged for a homeostatic signal in the hepatic portal system derived from increased intestinal gluconeogenesis from propionate which involves induction of gluconeogenic genes by butyrate.^45,46^ Given the concomitant exposure of the epithelium to butyrate and propionate, this mechanism suggests an elegant homeostatic nutrient sensor. In addition, increased propionate flux through the liver has been shown to reduce intrahepatic triglyceride which is also likely to elicit an improvement in hepatic and whole-body glucose homeostasis.^47^ Furthermore, acetate has been linked to suppression of adipocyte lipolysis, thus reducing free fatty acid (FFA) flux to the liver mitigating against fatty liver induced deterioration in glucose homeostasis.^48,49^ Finally, elevated plasma acetate has been shown to be inversely related to plasma insulin levels.^50^ A mechanism to explain this effect involves improved insulin response in pancreatic β cells, mediated by FFAR2 which induces improved glucose control.^51,52^ Taken together, the evidence suggests that SCFA elicit effects on multiple tissues in a concerted action to improve intestinal, hepatic and whole-body glucose homeostasis.

In addition to the direct SCFA derived signal from the gut there are concomitant signals, generated by primary SCFA production in the gut lumen. Gut hormones produced by the enteroendocrine cells in the colonic epithelium also exert beneficial effects on glucose homeostasis. The mechanisms of gut hormone driven effects on glucose homeostasis have been comprehensively reviewed elsewhere^53,54^ and are beyond the scope of this review. However, the production of SCFA by the microbiota in the gut lumen is an important initiating event for the gut-hormone derived signal.^55,56^

## SCFA effects on lipid metabolism

SCFA elicit effects on lipid metabolism and adipose tissue at several levels. In the liver, the fate of acetate is *de novo* lipogenesis (DNL) and cholesterogenesis, both of which appear to be inhibited by propionate.^57,58^ Thus the ratio propionate : acetate may be an important determinant of the contribution of colonic acetate to lipid stores. Recent work has also demonstrated that propionate alone is able to reduce visceral fat and liver fat.^47^ Increased circulating SCFA are associated with reduced adipocyte lipolysis and adipogenesis.^59^ SCFA also inhibit insulin stimulated lipid accumulation in adipocytes via FFAR 2 signaling, resulting in small more responsive adipocytes which is associated with reduced adipose inflammatory infiltrate.^60,61^ Acetate also appears to stimulate leptin secretion in adipocytes.^62^ Leptin is an important adipose derived homeostatic signal which regulates energy balance and appetite.^63^ The inhibition of adipose tissue lipolysis leads to reduced free FFA from the adipose tissue to the liver. In fatty liver disease, adipose derived FFA have been shown to contribute 60% of fatty acids to newly synthesized triglyceride in the liver while DNL contributes 26%.^64^ Rectal infusion of acetate and propionate has demonstrated a 40% reduction in serum FFA.^49^ The contribution of exogenous (gut microbiota derived) acetate production to whole-body acetate flux has been estimated to be approximately 44%^65^ but how this proportional contribution is affected by different NDCs and microbiome activity is largely unknown. Increasing peripheral SCFA availability from NDC fermentation may be a novel strategy to inducing regulation of FFA flux in the obese phenotype. However, controversy still exists regarding the role of SCFA in obesity. A number of studies have advanced the “energy harvesting” hypothesis, whereby SCFA are thought to contribute additional calories through fermentation in the obese as an explanation for additional weight gain.^66^ However, this is not supported by the observational evidence in humans where high-fiber diets, which would be expected to increase SCFA production, protect against weight gain.^67-69^ Further well controlled studies in humans are urgently needed to dissect the role of SCFA in lipid turnover and energy homeostasis.

## SCFA and appetite regulation

The role of SCFA in appetite regulation and energy intake has recently been reviewed in detail elsewhere.^70,71^ In addition to endocrine mediated effects, SCFAs can modulate neuronal activity and visceral reflexes directly via receptors expressed on neurons of the peripheral, autonomic and somatic nervous systems providing an additional mechanism of SCFA action.^72^ Whether these observations are driven by a single SCFA or a synergistic combination of SCFAs remains to be elucidated. In human intervention studies, only a small number of studies have demonstrated that fermentable fiber is associated with improved appetite regulation.^73-77^ Supplementation of habitual fiber intake in the range 16 – 35 g/d (approximately 5 – 10 % DMI) is necessary to induce these effects and may reflect the more consistent findings in animals at these equivalent fiber loadings and above. Whether these high fiber intakes elicit their appetite-regulating effects via SCFAs and FFAR 2/3 signaling pathways awaits a method to quantify SCFA production *in vivo*. Tantalising evidence of the role of SCFA in appetite regulation has recently appeared in a study using selective modulation of colonic propionate in humans which demonstrated that propionate appears to induce short-term appetite regulation though PYY and GLP-1 mediated mechanisms.^47^ Further work is needed to fully elucidate the role of each SCFA however.

## SCFA and immune function

An exciting area of recent investigation has arisen from the discovery that SCFA play a role in regulating the immune system and inflammatory response. Early work at the turn of this century had demonstrated the potential role of butyrate in immune regulation when it was shown that butyrate inhibits nuclear factor kappa β (NF-κΒ) activation in macrophages and also inhibits histone deacetylation (HDAc) in acute myeloid leukemia.^78,79^ NF-κΒ is a eukaryotic transcription factor that is involved in the control of a plethora of normal cellular processes, including immune and inflammatory responses. HDAc inhibition plays a role in specific inflammatory signaling pathways as well as epigenetic mechanisms.^80^ Recently, 2 studies have highlighted a potential role for propionate and butyrate in regulatory T cell production and function at the whole-animal level through inhibition of HDAc.^81,82^ The extra-thymic conditioning of regulatory T cell response by SCFA suggests that these molecules are an important link in the cross-talk between the microbiome and the immune system. Whether SCFA act as a signal to induce tolerance to the host-associated microbiome or directly reduce inflammatory responses remains to be fully elucidated. SCFA do appear able to reduce the responsiveness of lamina propria macrophages to commensal bacteria, via nitric oxide, IL-6, and IL-12 independent of FFAR signaling, to induce tolerance.^83^ SCFA, in particular propionate and butyrate, have also been shown to inhibit the expression of lipopolysaccharide (LPS)-induced cytokines, IL-6 and IL-12p40 in human mature dendritic cells.^84^ Of the few clinical studies that have used SCFA therapeutically in inflammatory disease in a controlled trial setting, improvements in clinical and histological indices of IBD and therapeutic efficacy in acute radiation proctitis have been observed supporting a direct anti-inflammatory role of butyrate at sites of inflammation.^85,86^ Decreases in butyrate-producing organisms have been observed in metabolic aberrations such as type-2 diabetes,^87^ which is a disease characterized by low-grade inflammation. Thus, an evolving body of evidence appears to support a crucial role for SCFA in shaping the local and peripheral immune system which impacts on host metabolism via inflammatory pathways.

The liver hosts a range of cell types that interact via small molecules and secondary immune cytokine signaling. Gut barrier permeability is thought to be a key factor in determining the pro-inflammatory load reaching the liver.^36^ Abrogation of hepatocyte triglyceride accumulation and fatty acid esterification, and decreasing fatty acid oxidation and insulin responsiveness has been observed in a murine Kupffer cell depletion model, largely mediated by TNF-α.^88^ Recent data also suggests butyrate suppresses TNF-α, IL-6, and myeloperoxidase activity by preventing NF-κΒ activation in Kupffer cells.^89^ There is a paucity of data regarding acetate and propionate, which would be present at higher flux through the liver. Although further evidence is required to establish the role of SCFA in regulating liver inflammation, either directly or indirectly, these studies demonstrate the importance of the gut-liver axis in inflammatory and metabolic systems and that SCFA may play important roles in both.

The role of SCFA also extends to peripheral immune function. A recent study has demonstrated that acetate mediates joint inflammation in a murine gout model through inflammasome assembly and IL-1β production that is partially FFAR2 dependent.^90^ A similar protective effect has been recently observed for butyrate in a peripheral blood mononuclear cell gout model, although high concentrations of butyrate were required to moderate production of the pro-inflammatory cytokines IL-1β, IL-6, IL-8 and IL-1β.^91^ Consideration of the biological gradient for SCFA exposure for different immune cell types may be critical to defining physiologically relevant outcomes in immune-mediated and inflammatory disease.

## Conclusions and future perspectives

**Figure 2.**
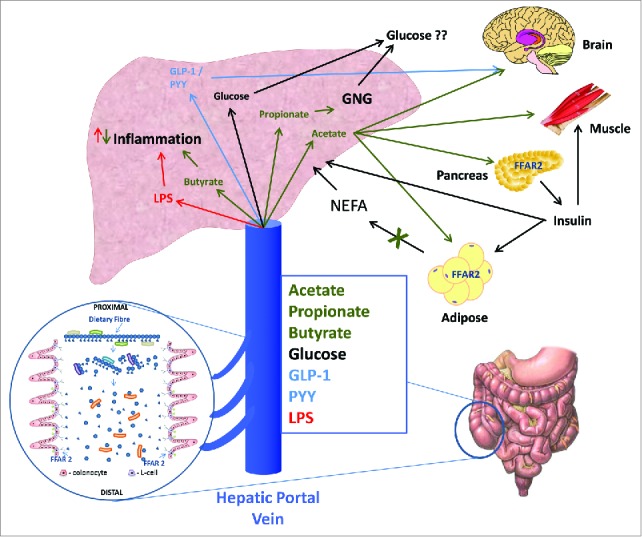
SCFA along with other metabolites entering the hepatic portal system are rapidly transported to the liver. The role of molecular signaling on different liver cell types is poorly characterized. SCFA can act on resident macrophages and hepatocytes although there may be functional selectivity for each SCFA. Incretion hormones may also act on hepatocytes and peripheral tissues. The overall impact of this dual signaling system appears to be maintenance of a healthy liver through regulation of hepatic metabolism and inflammation and control of adipose derived FFA flux. The peripheral effects of SCFA appear tissue specific. SCFA can regulate insulin in the pancreas, FFA flux from adipocytes, appetite centers in the brain and provide a fuel for the muscle. This multi-faceted role however, requires further investigation with well-designed and controlled studies in humans.

It is tempting to overlook the potential for small ubiquitous microbiota derived molecules like SCFA to act as important molecular signals between the microbiota and host or as metabolic substrates regulating host cellular metabolism. A continually emerging body of evidence supports the role of SCFA as key mediators of cell function in a range of local, intermediary and peripheral tissues (summarised in Fig. 2) raising the question as to whether SCFA represent *the* key molecular link between diet, the microbiome and health? The renewed interest in SCFA, coupled with the revolution in the tools available to dissect the complex molecular biology associated with cell signaling and metabolism, is beginning to provide evidence of the central role of SCFA in the diet-gut microbiome-host metabolism axis. There are however some key questions remaining that require further investigation. 1) *Is the microbiome a passive substrate-degrading system or are signaling molecules actively involved in microbiome-host “crosstalk”?* Undoubtedly the answer to this quandary is yes, the molecules produced through microbiota activity provide important regulatory signals to the host and in the “-omics” revolution, metabolomics is a key enabling technology which will enable the identification of the repertoire of signals between microbiome and host. 2) *Can the microbiota and its function be manipulated in a predictable way to have a clinically relevant impact on disease risk?* The answer to this is highly likely to be yes, but probiotic studies designed to change microbiota diversity have been far from convincing in terms of outcome measures in metabolic health^92^ and weight management.^93^ Prebiotics have had varying degrees of success and the lesson from animal feeding studies may be that high doses of NDC are needed in Western populations to drive physiologically relevant changes in SCFA in order to induce a physiologically relevant effect. This presents a major challenge to translating effective treatments into clinical practice or into strategies that improve population health. New targeted approaches may be needed. 3) *In Western societies and developing countries, what role do changes in diet and microbiome function play in future risk of disease?* There is an increasing focus on the prevention of diseases that cluster around obesity, inactivity and a Western-type diet because of the present and predicted future economic health burden. The multifaceted roles of SCFA suggest that they may play an important role over the life-course in protecting the body against deteriorating metabolic control and inflammatory status associated with Western lifestyles. Whether manipulating the diet-gut microbiome-host metabolism axis represents a panacea for prevention of these leading causes of morbidity and mortality remains to be seen but it is a tantalising prospect because of the wide ranging benefits that could be brought about through relatively simple and cost-effective interventions if they could be targeted appropriately.

## Abbreviations

*SCFA*: short chain fatty acids
*NDC*: non-digestible carbohydrates
*FFAR*: free fatty acid receptor
*IBD*: inflammatory bowel disease
*DMI*: dry matter intake
*UK NDNS*: United Kingdom national diet and nutrition survey
*TJP*: tight junction proteins
*LPS*: lipopolysaccharide
*TLR*: toll-like receptor
*NF-kΒ*: nuclear factor kappa β
*MAPK*: mitogen-activated protein kinase
*TNF-a*: tissue necrosis factor-α
*IL*: interleukin
*ZO-1*: Zonula Occludens-1
*FFA*: free fatty acid
*DNL*: *de novo* lipogenesis
*HDAc*: histone deacetylation
